# Development of a rapid detection technology system for noxious plants based on a novel isothermal amplification technique

**DOI:** 10.3389/fpls.2024.1502052

**Published:** 2025-01-20

**Authors:** Ting Zhang, Han Xu, Mengdi Liu, Wei Zhang

**Affiliations:** ^1^ Institute of Plant Inspection and Quarantine, Chinese Academy of Inspection and Quarantine, Beijing, China; ^2^ Marine College, Shandong University, Weihai, China

**Keywords:** noxious plants, isothermal amplification technique, rapid detection, ITS, seeds, mixed impurities

## Abstract

Noxious plants pose a significant threat to human and livestock health, as well as to the safety of agricultural and forestry ecosystems. Accurate and rapid identification of these plants is crucial for risk prevention. This paper explores for the first time the development and application of a rapid detection technology for noxious plants based on a novel isothermal amplification technique. We targeted the seeds, leaves, and grain impurities of four major noxious weeds: *Amaranthus palmeri*, the *A.tuberculatus* complex, *Rhaponticum repens*, and *Euphrosyne xanthiifolia*, we designed and screened primers and probes suitable for this isothermal amplification method, determined their limit of detection, optimized the genomic DNA extraction methods, and verified the method. We developed genomic DNA extraction methods for single tissue components of plant seeds and leaves, as well as for mixed tissue components. Ultimately, we established standardized detection protocols for different tissue forms of each species, significantly enhancing detection efficiency. This study enables the detection positive samples in seeds or leaves within 10 to 15 minutes and positive samples from mixtures within 12 to 18 minutes. The entire process, from sample collection, genomic DNA preparation to reaction completion, takes approximately 35 minutes. This detection technology, which marks the first development of an isothermal amplification-based method for noxious plants, meets the needs for on-site rapid testing, aiding in the timely identification of risks and the implementation of corresponding prevention and control measures.

## 1 Introduction

Noxious plants are widely distributed globally, and the natural toxins they contain can severely impact the health of humans and livestock (Fu et al., 2004; James et al., 1992), as well as the safety of agricultural and forestry ecological environments (Hulme et al., 2009). The globalization of trade has facilitated the long-distance spread of species (Hulme, 2009), which can be transmitted through seeds and other reproductive bodies, hitching a ride on plants and plant products. They can also exist in various forms of coarsely processed plant products for human and animal consumption, such as fragments and powders (Ward et al., 2013). Plant seeds mixed in plant products are small in size, diverse in morphological characteristics, and often lack distinctive features when they emerge as seedlings in the wild (Budiarto et al., 2021). Additionally, the plant tissue in coarsely processed plant products is often fragmented, all of the above factors make effective identification by morphological features difficult. Molecular biological methods such as DNA barcoding and qPCR require laboratory settings to be completed. Therefore, developing a method that can accurately and rapidly identify these noxious species on-site is crucial for preventing such risks.

*Amaranthus palmeri* S. Waston, *A. tuberculatus* (Moq.) J.D.Sauer, and *Euphrosyne xanthiifolia* (Nutt.) A. Gray are all native to North America, *Rhaponticum repens* (L.) Hidalgo is native to Western Asia. They are listed as invasive species by the Convention on the Protection of the Environment (CPC) and are under official control in several countries, posing threats to human and animal health as well as to agriculture and ecological environments (Follak, 2015; Iamonico, 2015; Schaffner, 2017; Vélez-Gavilán, 2019). The seeds of these plants are small, ranging from 1mm to 1cm, and are easily mixed with grains, fodder, and other plant products for long-distance transportation (Pyšek and Richardson, 2007), which has led to multiple interceptions at Chinese ports. Morphology is suitable for organisms with distinct and easily identifiable characteristics, but it loses its advantage when it comes to identifying seeds and seedlings with few diagnostic traits or species with high morphological variation. In current laboratory or field detection, there is an increasing reliance on molecular biology methods such as DNA barcoding (Xu et al., 2018), qPCR (Murphy et al., 2017), KASP (Brusa et al., 2021), and protein typing (Murphy et al., 2023), yet these methods all have their drawbacks in terms of time, procedural complexity, and contamination rates. For instance, DNA barcoding sequencing requires at least one day, while specific amplification fragments, qPCR and KASP take at least one hour, and are prone to contamination.

To meet the needs of rapid on-site detection, some researchers have applied isothermal nucleic acid amplification techniques such as LAMP (Notomi et al., 2000) and RPA (Piepenburg et al., 2006) in the detection of human, animal, and plant pathogens, as well as genetically modified organisms (Lobato and O’Sullivan, 2018; Wong et al., 2018). In the field of plant detection and identification, for instance, Focke (Focke et al., 2013) and Tian (Tian et al., 2017) used LAMP and RPA respectively to detect components in plant products, but the reference species they used were distantly related, which actually results in a problem of low resolution.

In recent years, many researchers have developed numerous new isothermal detection technologies by altering enzymes and other reagents, such as the isothermal amplification technique developed by Hu et al. (2024). Based on its excellent performance in the detection of African swine fever (Hu et al., 2024), such as high sensitivity and a zero contamination rate, we plan to use this technology in combination with the internal transcribed spacer (ITS) sequence to achieve rapid detection and identification of harmful plants. Building on our existing foundation of sample collection, species classification, and phylogenetic research on *A. palmeri*, the *A.tuberculatus* complex, *R. repens*, and *E. xanthiifolia* (Xu et al., 2020, 2022; 2024a; 2024b), we attempt to solve the challenges of detecting and identifying seeds, plant fragment tissues, and mixtures of plant products of these significant noxious plants on-site, on the premise of using closely related species as reference samples for these species, thereby enhancing species resolution.

The research primarily focuses on sample collection, sequence analysis, primer and probe design, method validation, optimization of plant nucleic acid preparation methods, and adaptation and optimization with isothermal detection technology systems. The development of this method fills the gap in on-site rapid detection technology for noxious plants in the fields of plant product quality safety, food safety, agricultural plant protection, and border port plant quarantine, guiding frontline technical personnel in the detection and identification of noxious species.

## 2 Materials and methods

### 2.1 Plant materials

The samples required for the experiment were sourced from field collection and interception at ports, with the collected samples approved by customs and other plant quarantine authorities. They comply with the Biosafety Law of the People’s Republic of China (Order of the President of the People’s Republic of China, No. 56) and ISPM 09: Guidelines for Pest Eradication Programs adopted by the International Plant Protection Convention. Habitats and biodiversity were not damaged, and endangered species were not involved. The document includes a total of 35 species and 65 samples, which belong to *A. palmeri*, the *A. tuberculatus* complex, *R. repens*, and *E. xanthiifolia*, as well as their respective closely related taxa. The samples are stored in the Plant Specimen Room of the Chinese Academy of Inspection and Quarantine (CAIQP) in Beijing, China (see Appendix Table S1). Different tissues of the same sample may be used for different experiments, as detailed in Appendix 1. Abbreviations for species are provided in Appendix 1.

### 2.2 DNA extraction

The experiment utilized two methods for genomic DNA extraction. One method involved the use of the Plant Genomic DNA Kit (Tiangen Biotech Co., China) for extraction. The plant tissue used for gDNA extraction was silica gel-dried leaves, approximately 10 milligrams in total, which were frozen with liquid nitrogen and then ground into a powder for 1 minute (1800 r·min^−1^) using a grinder (Retsch MM400, Germany). The powder was employed for gDNA extraction with the kit. The extracted gDNA were primarily used for primer and probe selection experiments.

The alternative method involved direct extraction using a lysis solution from Beijing Tsingke Biotech Co., Ltd. Approximately 1mm^3^ of seeds or leaf tissue was placed into the lysis buffer, and after appropriate treatment with specific lysis temperature and duration, the sample was diluted 100-fold and made ready for detection. It could be stored at -4°C for up to one week. The lysis buffer treatment method was mainly used for gDNA extraction optimization experiments and on-site validation experiments.

### 2.3 Optimization of genomic DNA extraction method

To better adapt to on-site rapid testing, we optimized the lysis conditions for plant tissue. For seeds and leaf tissues, approximately 1 mm^3^ of tissue containing endosperm was taken and added to about 50μl of lysis buffer. The seeds were moderately crushed before the addition of the lysis buffer to ensure that the lysis buffer could come into full contact with more tissue cells; leaves could be moderately crushed with a pipette tip after the addition of the lysis buffer. For both seeds and leaves, a gradient of lysis temperatures ranging from room temperature (RT) to 36.5°C, 50°C, 65°C, 80°C, and up to 95°C was set, with a total of six temperature gradients. For each temperature condition, a gradient of lysis times was set, including 30 seconds, 1 minute, 1 minute 30 seconds, 2 minutes, 3 minutes, 5 minutes, 8 minutes, and 10 minutes, totaling eight time-gradients.

Considering the complexity of the composition of mixed samples, for mixed samples, we set up three gradients of lysis temperatures at 65°C, 80°C, and 95°C, and for each temperature condition, we set lysis times of 30 seconds, 1 minute, 1 minute 30 seconds, 2 minutes, 3 minutes, 5 minutes, 8 minutes, and 10 minutes. The mixed samples used for lysis are approximately 0.3 cm^3^ and should be moderately crushed beforehand, after which they are added to a lysis buffer with twice their volume.

To verify the effective extraction of nucleic acids from target species, the experiment also designs specific primers based on the ITS sequence for each target species and performs specific sequence amplification on each lysate product tested. The method that can stably obtain the nucleic acids of the target species in the shortest time and at the lowest temperature will be selected as the optimal lysis scheme. The primer design method and software used are the same as in section 2.4.

### 2.4 Primer and probe design

For *A. palmeri*, *A. tuberculatus* complex, *R. repens*, and *E. xanthiifolia*, the ITS sequence was used for primer and probe design and screening. In addition to the ITS sequences obtained from laboratory sample sequencing, all sequences with a similarity of greater than 90% to these species were retrieved from the GenBank database for analysis. For *A. palmeri* and its closely related species, a total of 390 sequences were analyzed, with 31 sequences from 16 species obtained through sequencing and 359 sequences retrieved from GenBank; for the *A. tuberculatus* complex, and their closely related species, a total of 403 sequences were analyzed, with 43 sequences from 16 species obtained through sequencing and 360 sequences retrieved from GenBank; for *R.repens* and its closely related species, a total of 455 sequences were analyzed, with 12 sequences from 10 species obtained through sequencing and 443 sequences retrieved from GenBank; for *E.xanthiifolia* and its closely related species, a total of 261 sequences were analyzed, with 13 sequences from 9 species obtained through sequencing and 248 sequences retrieved from GenBank.

Subsequently, a multiple sequence alignment was performed for each species and its closely related species using Geneious alignment (Geneious version 2024.0.5), resulting in a neat matrix. SNP sites were identified and recorded, and specific regions were used to design 5~7 couples of DNA primers and 5~7 RNA primer probes for each target species with Primer 3 2.3.7 (see **Appendix Table S2–5**).

### 2.5 Primer and probe screening

#### 2.5.1 Reaction system and procedure

Fluorescent isothermal amplification detection kits were purchased from Suzhou Jingrui Biotechnology Co., Ltd. The kit consists of prepared enzyme dry powder (endonuclease, dNTPs, polymerase) and activation solution (reaction buffer, dNTPs, magnesium acetate) [for details, see literature, Hu et al. (2024)]. Primers were synthesized by Sangon Biotech (Beijing) Co., Ltd., and RNA primer probes were synthesized by Suzhou Jingrui Biotechnology Co., Ltd. Each reaction system is composed of enzyme dry powder (approximately 20μl in volume), upstream DNA primer (10μM) 1μl, downstream DNA primer (10μM) 1μl, RNA primer probe (1μM) 1μl, DNA template 7μl, and activation solution 10μl.

After the system is configured, it is centrifuged at 12000rpm for 30 seconds, mixed by shaking, and then detected on the machine. Under conditions of 42°C, fluorescence signal detection is carried out using a constant temperature fluorescence detector (Suzhou Jingrui Biotechnology Co., Ltd., uReader 1600). After the program is completed, data processing is performed, and the determination is made based on the time when the fluorescence signal exceeds the set threshold (threshold time). Based on Hu et al. (2024), a positive judgment standard is set when the fluorescence increase is greater than or equal to 200,000, and the time when the amplification fluorescence value exceeds 200,000 is defined as the threshold time.

#### 2.5.2 Preliminary screening of RNA primer probes

For positive samples, we randomly select two pairs of candidate DNA primers (e.g., F1R1, F1R2 or F1R2, F2R3) and conduct combination testing with all RNA primer probes. Each experiment is repeated three times, and ultimately, the RNA primer probe with the smallest threshold time value and the highest endpoint fluorescence value is selected for the screening of upstream and downstream DNA primers.

#### 2.5.3 Preliminary screening of DNA primers

Using the selected RNA primer probe, all candidate DNA primers are screened for primer specificity in a combinatorial manner. The samples used are positive samples and their closely related species (negative samples). Ultimately, the combination of DNA primers with the smallest threshold time value, the highest endpoint fluorescence value, and specificity is chosen.

#### 2.5.4 Final selection of DNA primers and RNA primer probe combinations

Using the DNA primer pairs selected from the screening in section 2.5.3, re-evaluate all RNA primer probes for sensitivity and specificity through experimentation. Ultimately, select the combination of DNA primers and RNA primer probes with the smallest threshold time value, the highest endpoint fluorescence value, and good specificity for subsequent experiments. The samples used are positive samples and their closely related species.

### 2.6 Limit of detection

Using the final selection of upstream and downstream DNA primers and RNA primer probe combinations, detect positive samples with different concentration gradients. The samples are nucleic acids extracted using the kit and measured for gDNA concentration with an ultramicroscopic spectrophotometer (NanoDrop ND-1000 Spectrophotometer), repeated three times, and the results are averaged. After determining the concentration, the nucleic acid samples are diluted by factors of 10, 100, 1000, and 10,000, and the gDNA concentration is measured again, with each measurement repeated three times. By combining the reaction curves and threshold time values, the detection limit of this method is determined.

### 2.7 Method verification

For each target species and its closely related taxa, as well as their various plant tissue forms, we tested blind samples using the selected compatible lysis methods, DNA primers, and RNA probe combinations. Each species has three types of samples: seeds, leaves, and mixtures, with 10 samples of each type, including 1 to 3 positive samples. There are a total of 120 blind samples for four species. The method verification includes both positive and negative controls. Ultimately, a detection protocol is established for different species and various plant tissue forms.

## 3 Results

### 3.1 Primers and probes screening results

**Figure 1:**
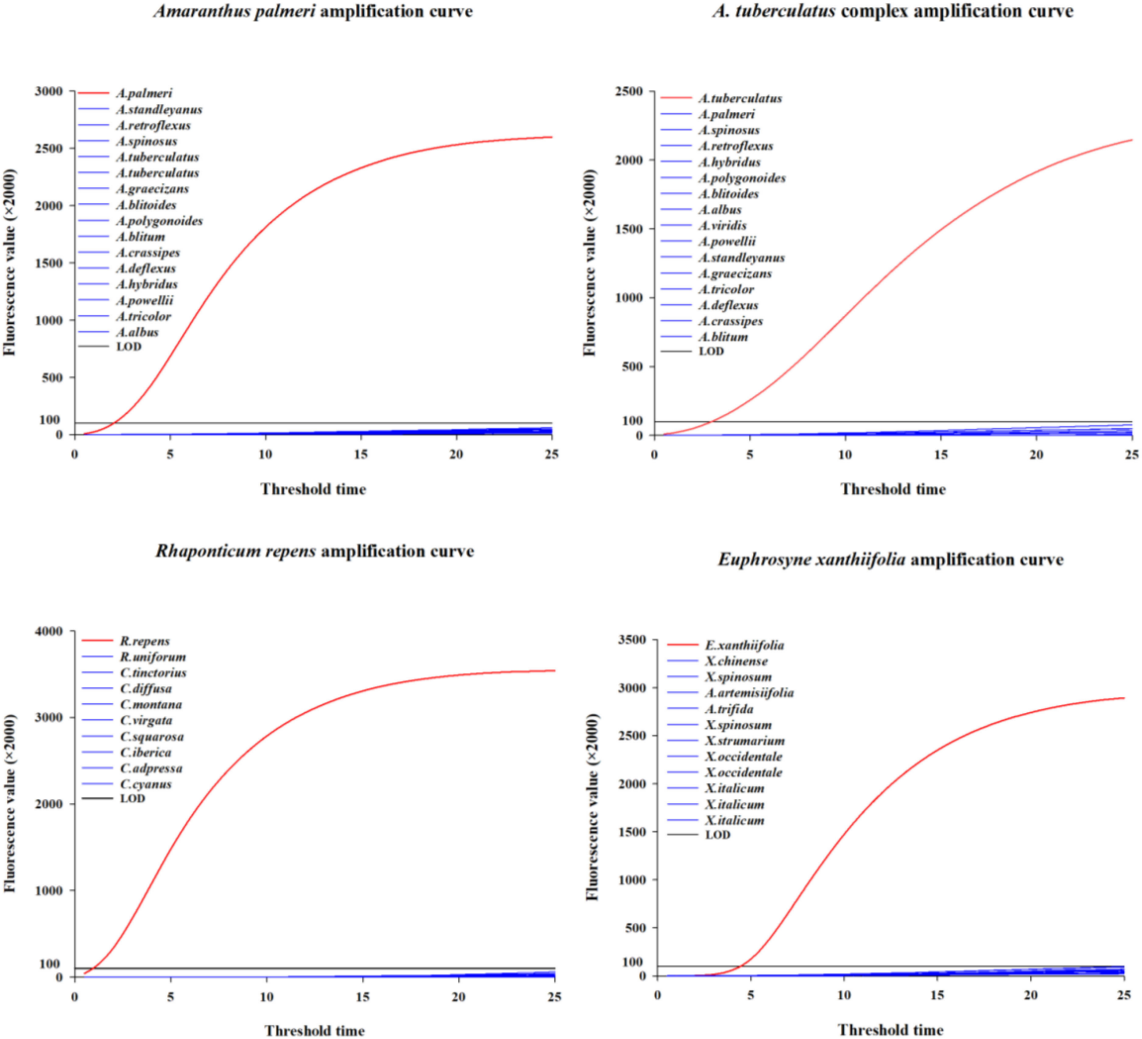
The specificity and sensitivity screening curves corresponding to the four species.

Based on the ITS sequences of *A. palmeri*, *A. tuberculatus* complex, *R. repens*, and *E. xanthiifolia*, 5-7 pairs of upstream and downstream DNA primers and 5-7 RNA primer probes were designed for each species (see Appendix Table S2-5). After specificity and sensitivity testing, a set of DNA primers and RNA primer probe combinations was ultimately selected for each species. The combinations of DNA primers, RNA primer probes, and target amplicon sequences are presented in **Table 1**. The species-specific and sensitivity test curves are shown in **Figure 1**.

**Table 1:** The final selection of primer combinations for the four tested species.

Species	Primer F	Primer R	RNA prime-probe	Sequence	Length
*Amaranthus palmeri*	PAL-F4: AAGCTAATACGACTCACTATAGGGGCCGGGCGTGGATGGCCTAAAAAG	PAL-R3: TCAAGGCCACAAGGTCCACGCTCTGTGC	PAL-A6: UUCUAGGCUAGGCCUUGCACACCACCAA	GGGCGAGGAGGATGGTCTCCCATGCCTCGCCGGG-CGTGGATGGCCTAAAA **AG** GGAGCCCG	70bp
*A. tuberculatus* complex	TUB-F3: AAGCTAATACGACTCACTATAGGGAGCCTAGAATGCAATCGCGTCGTACAGC	TUB-R4: AAACTCAGCGGGTAGTCCCGCCTGACCT	TUB-A3: GCAACACUCUAGGGUCCUCAAGGCCACA	GAGCTGCTGCGGCGATTGGTGGTGTGCAAGGCCTAGCCTAGAATGCAATCGCGTCG **T** ACAG **C** GCG	65bp
*Rhaponticum repens*	REP-F5: AAGCTAATACGACTCACTATAGGGCACGTCTGCCTGGGCGTCACGCATCGCGTCGT	REP-R3: AAAGGGGACTCCTTTTTAGGCCAACCAT	REP-A3: CCGUCCCAGACAAAACACAUCCCCAUGG	TCCCGTGCCTACGGC **AT** GGTTGGCCTAAAAAGGAGTCCCCTTTGGCGGGC	68bp
*Euphrosyne xanthiifolia*	XAN-F4: AAGCTAATACGACTCACTATAGGGATCATCGCAAGACAACGCGTTTGGGTCA	XAN-R4: TTGGGGCGGAGATTGGTCTCCCGTGCCC	XAN-A4: CGCACGACUAGUGGUGGUUGAUAACACA	AGATACTCTTAAA **TG** ACCC **A** AACGCGTTGTCTTGCGATGATGCTTCGATCGCGACCCCAGGTC	116bp

### 3.2 Limit of detection

**Figure 2:**
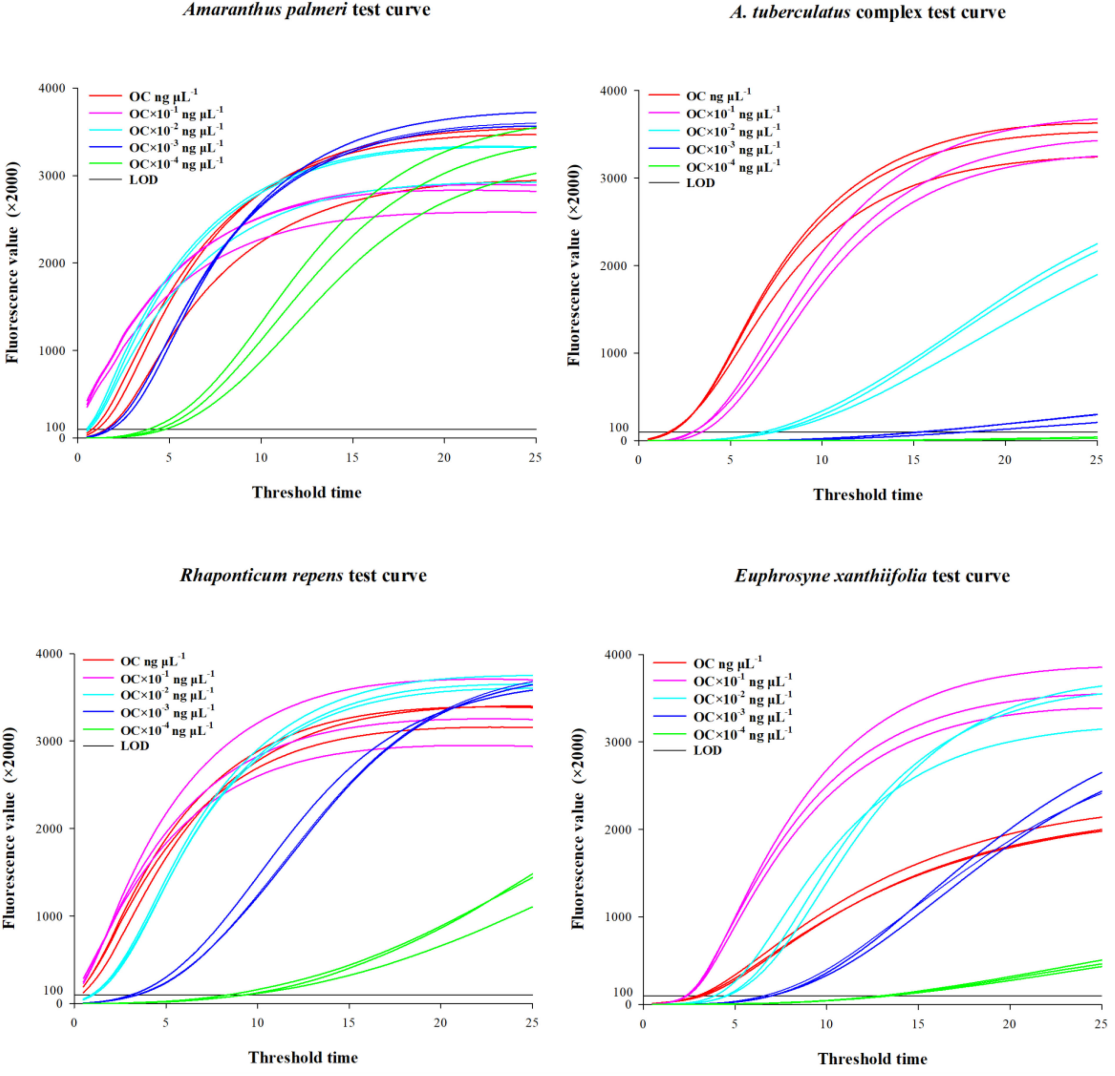
Amplification curves obtained from limit of detection testing.

The genomic DNA concentrations of the species used for testing were as follows: *A. palmeri* 46.4 ± 0.92 ng/μl, the *A. tuberculatus* complex 37.6 ± 0.61 ng/μl, *R. repens* 17.3 ± 0.29 ng/μl, and *E. xanthiifolia* 46.4 ± 0.30 ng/μl. The gDNA concentrations after dilution by factors of 10, 100, 1000, and 10,000, as well as the threshold time values when the fluorescence amplification reached or exceeded 200,000 for the five concentrations, are presented in **Table 2**, with the amplification curves shown in **Figure 2**. Based on the test results, it can be determined that *A. palmeri*, the *A. tuberculatus* complex, *R. repens*, and *E. xanthiifolia* can still detect the target nucleic acid fragments under conditions where the original gDNA concentration is diluted 1000-fold (to less than 0.1 ng/μl) (**Table 2**). However, the detection sensitivity varies with different species and concentrations. *A. palmeri* exhibits a lower threshold time when diluted 10-fold and 100-fold, corresponding to gDNA concentrations between 0.2 to 4.6 ng/μl, indicating higher detection sensitivity. For *R. repens*, the threshold time is less than 1 when the gDNA concentration is between 0.1 to 17.3 ng/μl, and the sensitivity decreases as the gDNA concentration further decreases. *E. xanthiifolia* has the smallest threshold time at a gDNA concentration of approximately 4.6 ng/μl. In contrast, the *A. tuberculatus* complex shows the smallest threshold time at the original gDNA concentration of 37.6 ng/μl, which increases with dilution, and the target fragment cannot be detected when diluted to 10,000-fold.

**Table 2:** Threshold time values for target species samples at various concentrations during constant temperature amplification.

Species	*Amaranthus palmeri*	*A. tuberculatus* complex	*Rhaponticum repens*	*Euphrosyne xanthiifolia*
Original concentration (OC) (ng/μl)	46.40 ± 1.61	37.67 ± 1.07	17.30 ± 0.30	46.43 ± 0.51
10-fold dilution (10F) (ng/μl)	4.50 ± 0.35	4.40 ± 0.35	2.13 ± 0.12	4.67 ± 0.23
100-fold dilution (100F) (ng/μl)	0.23 ± 0.15	0.53 ± 0.21	0.33 ± 0.15	0.37 ± 0.40
1000-fold dilution (1000F) (ng/μl)	-0.27 ± 0.23	-0.13 ± 0.25	-0.07 ± 0.25	0.27 ± 0.38
10000-fold dilution (10000F) (ng/μl)	-0.67 ± 0.49	-0.33 ± 0.15	0.00 ± 0.35	-0.40 ± 0.50
OC threshold time	1.07 ± 0.40	1.67 ± 0.06	0.10 ± 0.00	3.07 ± 0.15
10F threshold time	0.23 ± 0.06	3.07 ± 0.29	0.17 ± 0.06	2.27 ± 0.06
100F threshold time	0.37 ± 0.23	7.13 ± 0.31	0.73 ± 0.23	4.30 ± 0.35
1000F threshold time	1.60 ± 0.10	16.33 ± 1.45	3.27 ± 0.15	6.87 ± 0.23
10000F threshold time	4.27 ± 0.45	0.00 ± 0.00	9.00 ± 0.52	13.27 ± 0.29

### 3.3 Optimization of sample nucleic acid extraction temperature and time

**Figure 3:**
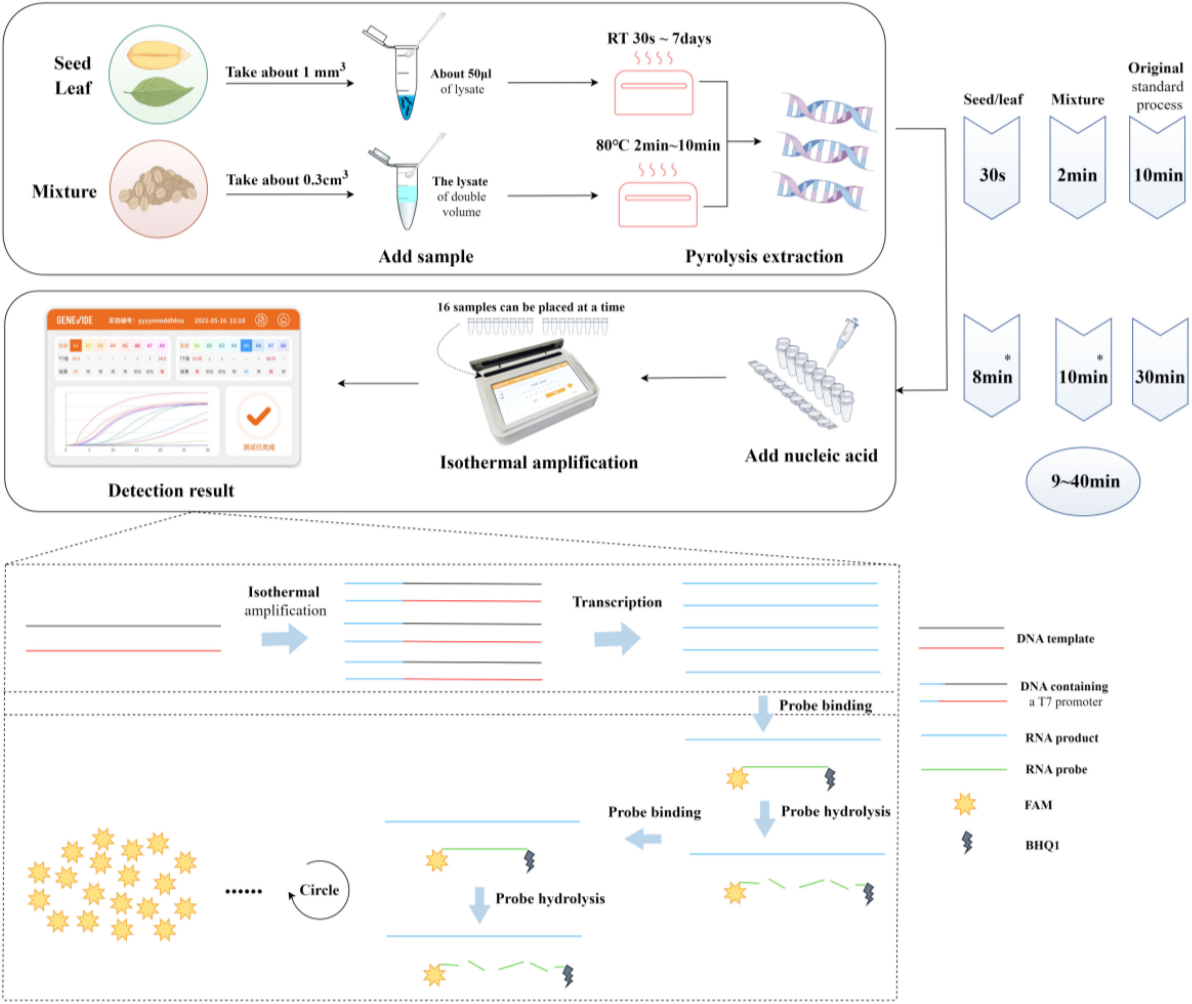
Integrated detection system flowchart. Create figure by Figdraw. *The duration indicated is the time for positive detection, not the completion of the reaction; the entire reaction is completed in approximately 30 minutes.

For each target species, the specific primers designed within the ITS sequence are presented in **Table 3**. For seeds and leaf tissues, lysis is performed at temperatures ranging from room temperature to 36.5°C, 50°C, 65°C, 80°C, and up to 95°C for 30 seconds, 1 minute, 1 minute 30 seconds, 2 minutes, 3 minutes, 5 minutes, 8 minutes, and10 minutes, respectively. The test results indicate that effective nucleic acid sequences of the target species can be obtained under all conditions (Sequence Information: GenBank Accession Numbers PP980754-PP980832, PP980834-PP980954, PP981480-PP981505). Based on the selection criteria, we prefer a lysis protocol that uses room temperature conditions for at least 30 seconds, which can effectively acquire the target species’ gDNA (**Figure 3**). Compared to the standard protocol of heating at 95°C for 10 minutes, this finding can reduce the lysis time by at least 9 minutes.

**Table 3:** Specific primers designed for 4 species.

	Name	F	R	Amplification length
1	*Amaranthus palmeri*	CGAGCTATTGCACCCTCCTC	GCTCCCTTTTTAGGCCATCC	434bp
2	*A. tuberculatus* complex	CCTTACGGACGAGCTGTTGC	CTGTACGACGCGATTGCATT	517bp
3	*Rhaponticum repens*	GCAGAACAACCCGTGAACAT	TCCTTTTTAGGCCAACCATGC	548bp
4	*Euphrosyne xanthiifolia*	CCTGGGCGTCACGTATCAC	GACAACGCGTTTGGGTCATT	224bp

For mixed samples, lysis was performed at three temperature gradients of 65°C, 80°C, and 95°C for 1min, 2min, 4min, 6min, 8min, and 10min, respectively. The test results indicated that at 80°C and 95°C, heating for 2 to 10 minutes allowed for stable nucleic acid extraction (Sequence Information: GenBank Accession PP980754-PP980832, PP980834-PP980954, PP981480-PP981505). However, results were not stable when heating for 1 minute or at 65°C for 1 to 10 minutes, with occasional failures in nucleic acid extraction (see Appendix Table S6). Based on the selection criteria, we prefer a lysis protocol using room temperature conditions for at least 30 seconds when on-site, which can effectively obtain the target species’ gDNA (**Figure 3**). Compared to the standard protocol of heating at 95°C for 10 minutes, this finding can reduce the lysis time by at least 8 minutes.

### 3.4 Method validation and protocol determination

**Figure 4:**
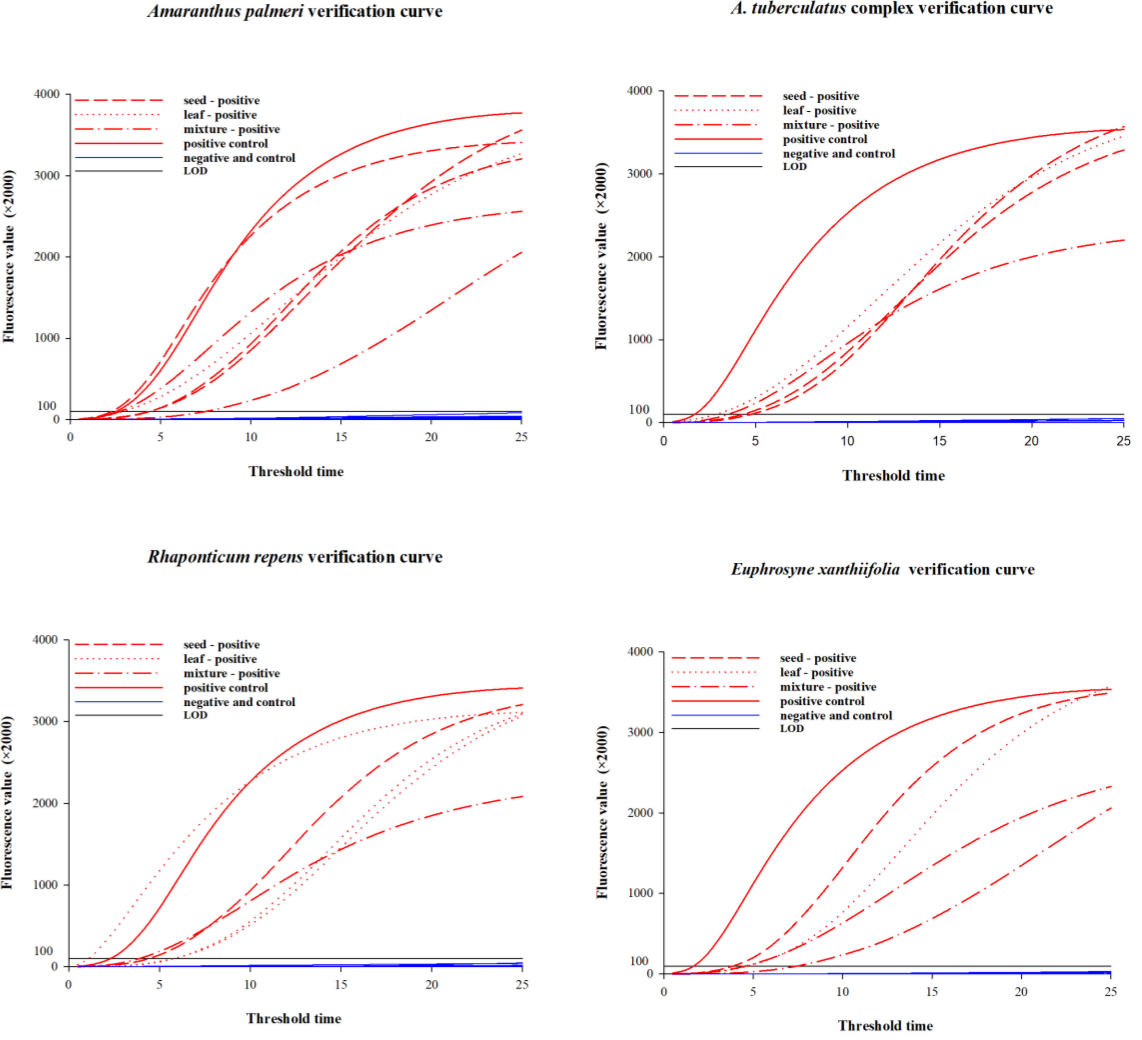
Amplification curves for samples of different tissue forms per species.

For four species and their closely related taxa, single seeds, leaf tissues, and mixed samples were subjected to method validation using the corresponding lysis methods and combinations of DNA primers and RNA primer probes (**Table 4**). The test results for samples of different tissue forms from each species are presented in **Table 4** and **Figure 4**. Among the 120 blind samples, the positive detection rate was 100%.

**Table 4:** Test combinations of samples with different tissue forms for each species.

Species	Lysis method		Primer-Probe Combination
	Single sample (seeds, leaves)	Mixed sample	
*Amaranthus palmeri*	RT 30s ~ 7 days	80°C~95°C 2min~10min	PAL-F4, PAL-R3, PAL-A6
*A. tuberculatus* complex			TUB-F3, TUB-R4, TUB-A3
*Rhaponticum repens*			REP-F5, REP-R3, REP-A3
*Euphrosyne xanthiifolia*			XAN-F4, XAN-R4, XAN-A4

## 4 Discussion

This study successfully developed and validated a rapid detection and identification technology system for noxious plants based on a novel isothermal amplification technique. This detection system enables rapid detection and identification of target plant tissues with gDNA concentrations below 0.1ng/μl on-site, such as in fields, wilderness, and cargo inspection ports.

The study is primarily based on solid taxonomic and phylogenetic research foundations of four noxious organisms. Building on previous work (Xu et al., 2020, 2022) and an ongoing study (Xu, 2024a; 2024b), the team has established the species classification and phylogenetic relationships of *A. palmeri*, the *A.tuberculatus* complex, *R. repens*, and *E. xanthiifolia*, laying the groundwork for the development of rapid isothermal amplification detection techniques for the four noxious plants involved in this study. In terms of specific sequence selection, the ITS sequence was found to have better specificity and conservation compared to the chloroplast genome and ALS fragments, and being multi-copy, it can achieve interspecific differentiation. However, for the polyphyletic hybrid complex like the *A.tuberculatus* complex, neither ITS, chloroplast genome, nor ALS can meet the identification requirements. Therefore, the experiment targeted the *A.tuberculatus* complex, actually addressing the detection and identification issues of *A. tuberculatus*, *A. arenicola*, *A. floridanus* (S.Watson) J.D.Sauer, and *A. greggii* S.Watson based on the ITS sequence. For the identification of the *A.tuberculatus* complex, additional collection of closely related species specimens and methods such as second-generation sequencing for Single Nucleotide Polymorphism (SNP) sites screening should be considered to discover more suitable SNP sites for the development of molecular detection techniques.

In the primer and probe screening phase following species sequence comparison analysis, both primers and probes affect the specificity and sensitivity of the detection. For instance, a primer-probe combination with good specificity may not necessarily have the best sensitivity. Conversely, the combination with the highest sensitivity may not meet the specificity requirements. Therefore, we designed numerous primer-probe combinations to satisfy the demands for both specificity and sensitivity.

In addition, during the detection limit experiment, due to the lowest measurement limit of existing gDNA concentration measurement equipment being 2ng/μl, the measured gDNA concentrations were not accurate when diluted 100 to 10,000 times. However, since the initial concentration of the gDNA extraction liquid was accurate, it does not affect the approximate estimation of the detection limit based on this method. However, the optimal detection concentrations vary among different species, which may be related to the inherent components that the species possess, as well as the degree of sequence binding with primers and probes. In the pre-treatment optimization experiment, the nucleic acids directly extracted with the lysis buffer contained a relatively high amount of original impurities, and the pH value of the lysis buffer itself could also affect the reaction efficiency of the enzyme preparation. Therefore, it is necessary to dilute the original lysis buffer by 10 or 100 times to ensure the normal function of the enzyme preparation.

Throughout the entire process from sampling, sample preparation, nucleic acid extraction to adding samples and running the detection, we have groped the shortest time and lowest temperature for nucleic acid preparation of plant seeds, leaves, and mixed samples, reducing the entire procedure by 8 to 9 minutes. In previous work, nucleic acid preparation often required a longer time, such as the need for conditions at 95°C for 10 minutes to obtain nucleic acids from dried samples. In the field environment, in order to achieve portable equipment and rapid detection, we try not to carry additional heating devices, such as metal baths, and also avoid the time cost of waiting for the equipment to heat up. Moreover, to simplify the operation process, in the future, the selected primer-probe combinations and enzyme preparations can be formed into freeze-dried powder, which can further reduce operation steps, such as direct sample addition and mixing, and then ready for detection. In summary, the rapid detection system for noxious plant seeds, leaves, and other single tissue components, or mixed samples, developed based on this novel isothermal amplification technology, fills the gap in on-site rapid detection technology for noxious plants in the fields of plant product quality safety, food safety, agricultural plant protection, and border port plant quarantine, guiding frontline technical personnel in the detection and identification of noxious species.

## Data Availability

The datasets presented in this study can be found in online repositories. The names of the repository/repositories and accession number(s) can be found in the article/**Supplementary Material**.
